# Designing Mobile Epidemic Prevention Medical Stations for the COVID-19 Pandemic and International Medical Aid

**DOI:** 10.3390/ijerph19169959

**Published:** 2022-08-12

**Authors:** Mi-Zuo Gao, Ying-Hsiang Chou, Yan-Zin Chang, Jar-Yuan Pai, Henry Bair, Sharon Pai, Nai-Chi Yu

**Affiliations:** 1 Institute of Medicine, Chung Shan Medical University, No. 110, Sec. 1, Jianguo N. Rd., South Dist., Taichung City 40201, Taiwan; 2 Radiotherapy, Department of Medical Imaging and Radiological Sciences, Chung Shan Medical University Hospital, Chung Shan Medical University, No. 110, Sec. 1, Jianguo N. Rd., South Dist., Taichung City 40201, Taiwan; 3 Department of Health Policy and Management, Chung Shan Medical University Hospital, Chung Shan Medical University, No. 110, Sec. 1, Jianguo N. Rd., South Dist., Taichung City 40202, Taiwan; 4 Byers Eye Institute, Department of Ophthalmology, Stanford University School of Medicine, 450 Jane Stanford Way, Stanford, CA 94305, USA; 5 Department of Health Science, University of Washington, 4218 Roosevelt Way, Seattle, WA 98105, USA

**Keywords:** epidemic prevention, mobile medical station, COVID-19, international medical aid, medical sustainability

## Abstract

The demand for mobile epidemic prevention medical stations originated from the rapid spread of the COVID-19 pandemic. In order to reduce the infection risk of medical practitioners and provide flexible medical facilities in response to the variable needs of the pandemic, this research aimed to design mobile medical stations for COVID-19 epidemic prevention, the emergence of which began in February 2020. The mobile medical stations include a negative pressure isolation ward, a positive pressure swabbing station, a fever clinic and a laboratory. In Taiwan, many medical institutions used the mobile swabbing station design of this study to practice COVID-19 screening pre-tests. Internationally, this study assisted Palau in setting up medical stations to provide anti-epidemic goods and materials. The design of this study not only provides a highly flexible and safe medical environment but the benefits of screening can also be used as resources for medical research, forming an economic circulation for operation sustainability. In addition, the design of this study can also be used during the non-epidemic period as a healthcare station for rural areas or as a long-term community medical station.

## 1. Introduction

The demand for mobile epidemic prevention medical stations originated from the rapid spread of the COVID-19 pandemic. An unidentified pneumonia cluster infection was discovered in Wuhan, China, in December 2019, and the nomenclature of the virus was named SARS-CoV-2 (Severe Acute Respiratory Syndrome Coronavirus 2) by the International Committee on Taxonomy of Viruses (ICTV). The virus spread rapidly across China and all over the world. People infected with the virus mostly experience mild to moderate respiratory illness, while some of old age or with underlying medical conditions such as cardiovascular disease, diabetes, chronic respiratory disease and cancer develop serious illnesses which require special medical care. SARS-CoV-2 is transmitted mainly through the inhalation of aerosols or droplets and also through interaction with contaminated surfaces [1,2]. Research showed that SARS-CoV-2 aerosols remain viable in the air for a duration of at least 3 h with an hour of half-life and are contagious to infect the human host [3]. According to the World Health Organization (WHO) COVID-19 dashboard, a total of 412,351,279 cumulative confirmed cases and 5,821,004 cumulative deaths were reported globally up to 16 February 2022 [4]. Within just two years, the highly contagious virus spread all over the world and constantly developed into several variants named Alpha, Beta, Gamma, Delta and Omicron. The impact of the COVID-19 pandemic not only threatens people’s health and lives but also affects every aspect of society.

Effective epidemic prevention principles and strategies come from the understanding of the virus and its transmission routes. According to WHO IPC (Infection Prevention and Control) precaution recommendations and current evidence, SARS-CoV-2 is transmitted between people through respiratory droplets, airborne and contact routes [5]. Droplet transmission, generally considered to be >5–10 µm in diameter, occurs when interacting with someone who has respiratory symptoms within 1 m social distancing and is therefore at risk of having mucosae or conjunctiva exposed to potentially infective droplets, whether through direct transmission or indirect contact with surfaces in the immediate environment and contaminated objects [6,7]. Different from droplet transmission, airborne transmission refers to the presence of microbes within droplet nuclei which are generally considered to be particles <5 µm in diameter resulting from the evaporation of larger droplets or exist within dust particles. The particles may remain in the air for long periods of time and be transmitted over distances greater than 1 m [8,9].

The most significant epidemic prevention policy agenda for the COVID-19 outbreak is to identify appropriate control measures to stop the chain of infection [10]. Nowadays, the infection of coronavirus is tested through Reverse Transcription-Polymerase Chain Reaction (RT-PCR) [11] and is considered a key response measure for COVID-19 [12]. Therefore, to stop the infection chain, screening centers serve as frontiers for preventing community transmission of infectious SARS-CoV-2. In South Korea, COVID-19 screening centers were operated 24 h a day for individuals with suspected COVID-19 symptoms. These emergency screening centers were located around cities with high population density. The major reason for South Korea’s success in flattening the curve of COVID-19 cases was their effective and efficient management of COVID-19 screening centers [13].

When it comes to public health policy, the dilemma of restrictive budget limitations for healthcare expenditures and a growing population have become even more important during the COVID-19 pandemic. One of the solutions to provide cost-effective primary healthcare in developing countries is mobile healthcare facilities, which can satisfy the most urgent needs and provide certain specialized medical divisions [14]. Similar mobile healthcare facilities have already been applied all around the world, especially in areas that lack medical resources. For instance, mobile health clinic programs [15] in the US play an important role in the healthcare system: providing healthcare access for displaced or isolated individuals; offering versatility in damaged or inadequate infrastructure; serving as a community-based service delivery model that fills gaps in the healthcare safety-net in urban and rural areas. A program of Harvard Medical School’s Family Van, the Mobile Healthcare Association and Mobile Health Map, demonstrated that the flexibility and adaptability of mobile clinics can make them an ideal partner in responding to the COVID-19 pandemic [16].

In order to respond to the medical needs of society, mobile epidemic prevention medical stations were designed and applied to the healthcare system in Taiwan. During the pandemic, it is essential to maintain daily medical capacity with limited resources while planning out strategies to stop the chain of infection at the same time. To cut off the virus transmission routes effectively, confirmed cases must be identified and quarantined in a short period of time. Thus, screening completed safely in an independent space has become the top priority. As the pandemic continues to heats up, it is relevant to increase the capacity of temporary swabbing stations and laboratories. Meanwhile, it is also necessary to pay attention to whether the number of dedicated isolation wards is sufficient enough to meet the demand. In this study, based on expertise in public health and the experience of patented technology, mobile epidemic prevention medical stations are designed by renovating and transforming containers into mobile multifunctional medical stations, including a negative pressure isolation ward, a fever clinic, a laboratory and a positive pressure swabbing room.

## 2. Methods

### 2.1. Design Concept: Mobility and Demands

**Figure 1:**
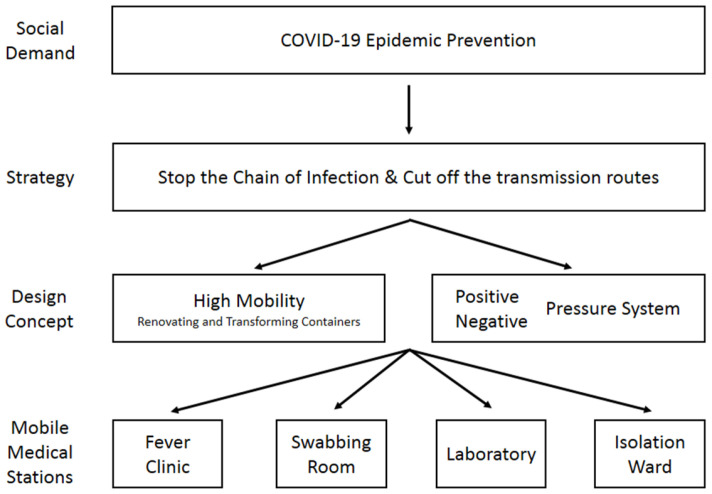
Concept used for the design of mobile medical stations.

**Figure 2:**
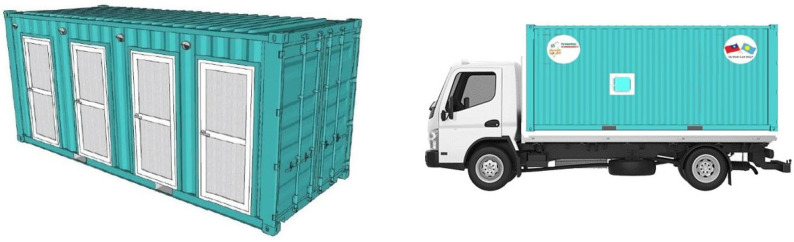
Simple schematic of the appearance of a mobile medical station.

Figure 1 shows the concept used for the design of mobile medical stations. The main concept of designing the medical station is to focus on the high mobility of its functions. In recent decades, mobile medical facilities have been deployed by governments and international organizations when disasters and wars occur. The advantages of a mobile facility are good mobility, strong adaptability to the environment, rapid deployment, complete medical functions, early treatment and so on. Mobile facilities can be categorized into three types: terrestrial, floating and flying. Among them, terrestrial mobile facilities are the most common type, including tent facilities, vehicle facilities and shelter facilities [17]. With high mobility, medical stations are able to support COVID-19 prevention effectively by adjusting the station setting as the occasion demands. The key point of roll planning prevention strategies is not being limited by space. Hence, mobile epidemic prevention medical stations are designed by renovating and transforming 40 ft and 20 ft containers into mobile multifunctional medical stations. The advantage of using containers as the main body is that containers can be moved easily compared to fixed buildings. The containers can be mounted on trucks which makes it easier to transport them to locations in need of medical attention. Figure 2 show a simple schematic of the appearance of a mobile medical station. In addition, the medical station can also be used during non-epidemic periods, e.g., as a mobile medical unit in a remote area [18]. Furthermore, it can be used as a first aid station or field operation room during emergency disasters and wars. Other than that, it can mostly be used as a healthcare station for rural areas and offshore islands or as a long-term community medical station. This flexibility means that the design of the containers should remain in line with the principle of maximizing the use of medical resources. Therefore, the containers should be able to meet the users’ demands and provide medical services under any circumstances.

### 2.2. Cut off the Transmission Routes: Positive and Negative Pressure System

Healthcare personnel should continue to maintain their right to have healthy and safe working conditions in the context of COVID-19 [19]. SARS-CoV-2 spreads rapidly through droplet and airborne transmission, which is imperceptible to the naked eye. Thus, the technology of negative and positive pressure plays an important role when it comes to designing medical facilities and protecting the safety of healthcare personnel from occupational exposure to infection risk [20]. Positive and negative pressure systems are common and indispensable facilities to control nosocomial infection in medical institutions. A positive pressure system enables the positive pressure ward area to maintain a stable pressure higher than the surrounding environment, which means that the air can leave the ward without backflow. In contrast, a negative pressure system provides lower air pressure to allow outside air to enter the isolation ward, preventing the interior contaminated air from leaving the area and filtering potentially harmful particles in the negative pressure room to protect others from exposure risk [21]. Therefore, a positive pressure swabbing room and a negative pressure isolation ward are suitable for airborne infection control.

### 2.3. Specifications and Standards

The specifications and standards are as follows:

1. The negative pressure ventilation system must be tested by SGS (General Surveillance Society; report no. EKR2004706), and measurement results should be compliant with the Taiwan CDC (Centers for Disease Control) standard operating instructions for negative pressure isolation wards.
2. The air-conditioning system must be able to switch between positive and negative pressure modes and be equipped with a HEPA (High-Efficiency Particulate Air) ventilation system. At the same time, the exhausted air must be sterilized at a high boiling temperature (100 °C).
3. The medical stations must be equipped with a UV lamp and O_3_ ozone sterilization.
4. Air-conditioning must be above 2.8 kW.
5. A solar panel of 100 Ah, power 5000 VA/5000 W and lithium battery above 2750 mAh must be used with an automatic generator for continuous operation.
6. There must be a differential pressure gauge and sensor.
7. Available 4G Wifi.
8. Firefighting installation devices must be certificated by the Taiwan Standard for Installation of Fire Safety Equipments Based on Use and Occupancy.
9. A CNS (Chinese National Standards-Taiwan) 14705 certified permit must be held for the cleanroom.
10. TFTA (Taiwan Food and Drug Administration) certified permits must be held for medical electric hospital beds and equipment.
11. A smart water heater and medical-grade air purifier must be present.
12. A Taiwan Ministry of Health and Welfare construction license for renovating containers into negative pressure isolation wards must be obtained.
13. A biosafety level 3 (BSL-3; P3) standard laboratory must be used.
14. The laboratory must possess a Taiwan Ministry of Economic Affairs IPO (Intellectual Property Office) patent certificate.

## 3. Results

### 3.1. The Contents of Mobile Epidemic Prevention Medical Stations

**Figure 3:**
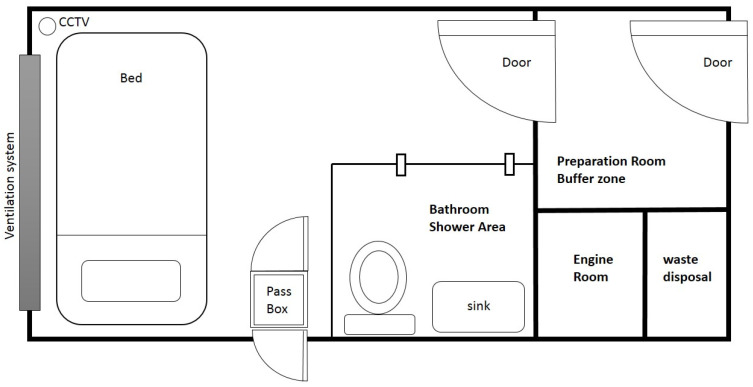
Layout of the negative pressure isolation ward.

The contents of mobile epidemic prevention medical stations include a negative pressure isolation ward, a positive pressure swabbing room, a fever clinic and a laboratory. The negative pressure isolation ward is equipped with a HEPA ventilation system, oxygen supply system, water supply and drainage system, solar energy storage and power system, negative pressure mode air-conditioning system and firefighting installation system. To isolate diagnosed patients and protect nurses and doctors [22], a negative pressure isolation ward is also designed with a preparation room, a bathroom and a waste matter disposal room. Figure 3 show the layout of the mobile negative pressure isolation ward. Other equipment can be included according to the demand of the users, such as a pass box, medical table and chair, stethoscope, bedside monitor, intubation first aid kit, CCTV or lockers.

**Figure 4:**
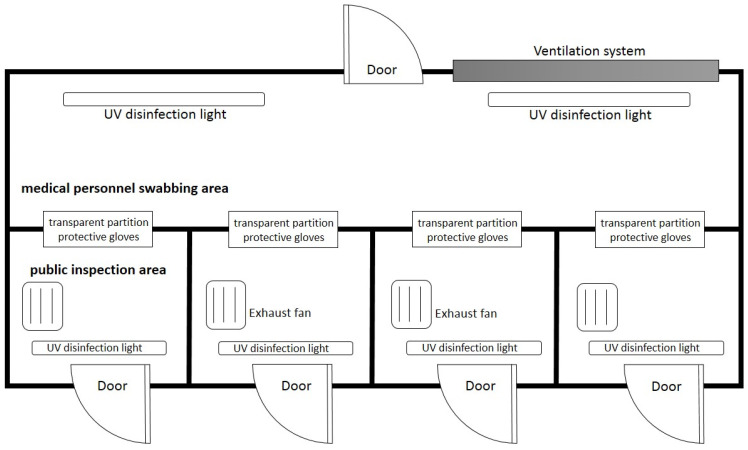
Layout of the positive pressure swabbing room.

The positive pressure swabbing room is equipped with a HEPA ventilation system [23], solar energy storage and power system, firefighting installation system and positive pressure mode air-conditioning system. To avoid infection risk, the positive swabbing room consists of a medical personnel swabbing area and a public inspection area with a transparent partition and protective gloves in between, forming a zero-contact swabbing space. The transparent partition and protective gloves are combined on the same plane to separate the two areas to form protection and avoid the risk of droplet infection or direct contact cross-infection during swabbing. Coupled with the function of the positive pressure system, medical personnel only need to wear masks when entering the container for inspection work and do not need not wear full-body protective clothing. Moreover, compared to traditional temporary swabbing stations set in open spaces such as community centers or public grounds, the design of this study sets a series of protective disinfection measures in each compartment of the public inspection area, including a medical waste disposal bag, an exhaust fan, a HEPA filter, an UV disinfection light and an auto-induction alcohol sprayer. Figure 4 show the layout of the positive pressure swabbing room. This design is both safe and comfortable, which not only protects the health of medical personnel and recipients but also enhances work efficiency while working in a comfortable environment with an air-conditioning system running.

**Figure 5:**
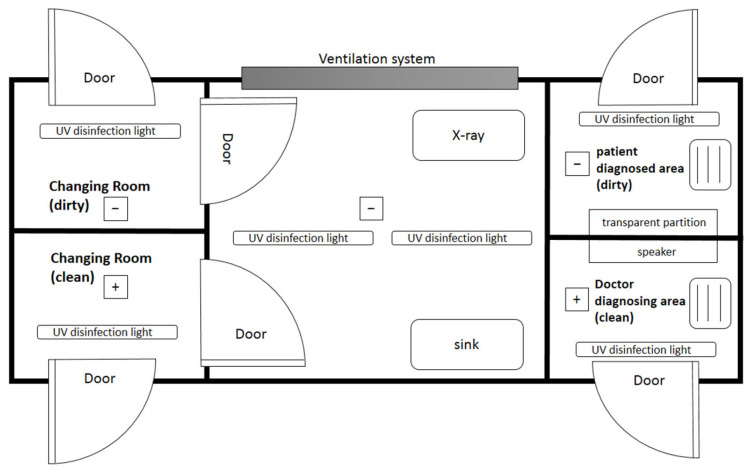
Layout of the fever clinic.

The fever clinic plays an important role in the early detection, isolation and referral of patients presenting with fevers of unknown origin [24]. As a dedicated space for the diagnosis and treatment of suspected COVID-19 patients, the fever clinic is equipped with a mobile digital X-ray system, lead screen, protective lead clothing, water bath and medical-grade air-purifier in addition to the usual equipment set in the general clinic. Ventilation systems and UV disinfection systems are also important to protect the safety of medical personnel. Figure 5 show the layout of the fever clinic.

**Figure 6:**
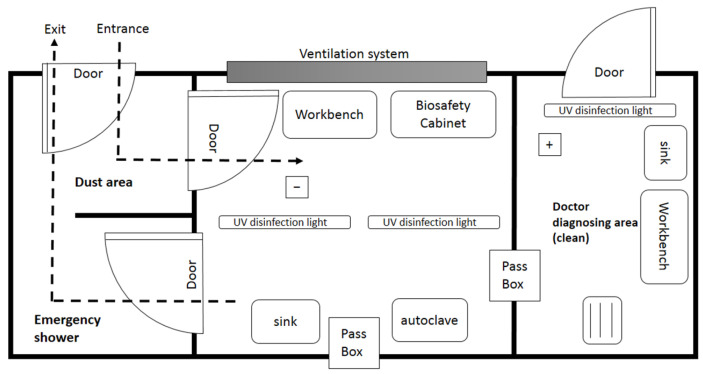
Layout of the mobile laboratory.

The laboratory is designed as a biosafety level 3 (BSL-3; P3) laboratory, a set of biocontainment precautions required to isolate dangerous biological agents in an enclosed facility [25,26]. Biosafety is based on the principles of containment and risk assessment, referring to safety methods used to manage infectious materials in a laboratory environment to protect laboratory workers, others outside the laboratory and the external environment from exposure to potentially hazardous agents [27]. The laboratory consists of an emergency shower area, dust area, entrance buffer area, independent laboratory space and biological safety cabinet operating device. A ventilation system and UV disinfection equipment that meet the BSL-3 laboratory standards are also set up. Figure 6 show the layout of the mobile laboratory. Other laboratory equipment can be configured as required, such as an ultra-low-temperature freezer, centrifuge, autoclave, water bath, ice maker machine, thermo mixer incubator, pipette, etc. 

### 3.2. Practical Experience of Mobile Epidemic Prevention Medical Stations in Taiwan

**Figure 7:**
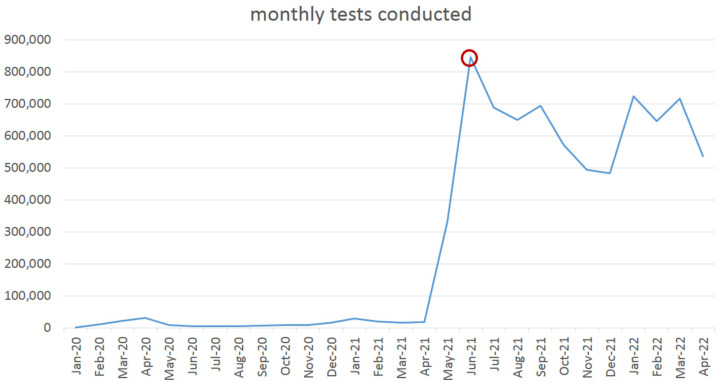
The line chart of monthly tests conducted in Taiwan. Data sources: Taiwan CDC.

In Taiwan, mobile epidemic prevention medical stations are mostly used as positive pressure swabbing rooms. According to Taiwan CDC data, a total of 7,591,630 PCR tests were conducted up to 14 April 2022 [28]. During this time, hospitals located in Taipei City, Taichung City, Changhua County and city public health centers of Tainan, Kaohsiung and Pingtung employed the positive pressure swabbing rooms proposed in this study which played an important role in increasing the screening capacity of medical facilities. During the outbreak on May 2021 in Taiwan, Taoyuan General Hospital of the Ministry of Health and Welfare was urgently in need of a large number of screening pre-tests in a short period of time and therefore used the mobile positive pressure swabbing room proposed in this study, dedicated to assisting the hospital in coping with the rapidly changing situation. Figure 7 is the line chart of monthly tests conducted which shows that the demand for screening reached its highest peak after the outbreak in May 2021. Swabbing stations are set outside hospital buildings to avoid the possibility of nosocomial infection. Each container is renovated into a four-inspection window swabbing station, which means four specimens can be collected simultaneously in 2–5 min with the disinfection process included in the swabbing procedure. Therefore, with four inspection windows operating at the same time, a swabbing station can collect 48–120 specimens per hour. The difference in the number of specimens collected depends on the medical personnel’s familiarity with the swabbing procedure and the cooperation of the subjects. The partition of the upper and lower structure in the container is made of steel, which is resistant to collision during transportation and is not easily deformed or broken. The partition insulates heat and regulates temperature, which makes this structure suitable for the hot summer climate in Taiwan.

### 3.3. International Medical Aid in Palau

Humanitarian aid covers a broad range of activities, including emergency relief delivered to people struck by natural or man-made disasters [29]. Among them, international medical aid is an important part of international humanitarian aid, which is recognized as a universal value that crosses through races, classes, politics, wars and cultures. This study co-operated with the Taiwan Ministry of Foreign Affairs and the Embassy of Taiwan in the Republic of Palau. While relatively isolated, the interconnectedness of air travel networks shows that the Pacific Island countries and territories are vulnerable to global disease threats [30]. The islands of Palau are geographically isolated and are considered medically underserved [31]. Therefore, the international medical aid project was activated to assist Palau during the COVID-19 pandemic in May 2020. A total of six mobile epidemic prevention medical stations were delivered and set up, including two mobile negative pressure isolation wards, one mobile fever clinic, one mobile positive pressure swabbing room and two mobile laboratories. The positive pressure swabbing station was set up at the airport to screen passengers passing through immigration. In January 2022, the epidemic in Palau encountered a severe challenge and reached the highest peak ever since the pandemic started globally; more than 500 confirmed cases were reported. The mobile medical stations came in handy during the Omicron outbreak, allowing diagnosis, treatment and screening work to be executed safely.

The installation of the medical stations must meet local demands and conditions. Due to the lack of tap water systems in Palau, the construction must include a water collector on the roof and a water pumping motor. As for the current situation of an unstable power supply, a gasoline engine generator must also be prepared in case of emergency. In order to ensure the strictness of epidemic prevention and prevent quarantined patients from leaving the mobile isolation wards and entering the community without authorization, the negative pressure isolation ward requires a locking design from the outside. In addition, for safety reasons, a warning light should also be installed outside the isolation ward. Whenever there is human activity inside the ward, the warning light will be activated to prevent people from being accidentally locked into the ward by mistake. 

## 4. Discussion

### 4.1. Maximizing the Use of Medical Resources with Flexibility

During the early stages of the COVID-19 outbreak, governments were required to make critical decisions on how to respond, despite limited data being available [32]. In this situation full of uncertainties, maximizing the use of limited medical resources is the priority principle of planning prevention strategies. In Taiwan, negative pressure isolation wards are not standard facilities in all hospitals. Although the standards of setting up medical institutions promulgated by the Ministry of Health and Welfare state that isolation wards can be set in hospitals and planned as general isolation wards, negative pressure isolation wards and positive pressure isolation wards, there is no clear specification for the number of isolation wards being set. In other words, the negative pressure isolation ward is a selective setting which will encounter deficiencies when treating a large number of patients with airborne infections in an outbreak. If medical institutions set up additional negative pressure wards in response to the COVID-19 outbreak, the facilities may become idle after the pandemic eases, resulting in a waste of medical resources. The design of a mobile negative pressure isolation ward can solve the problem mentioned above. Not only can patients be properly cared for and prevent the spread of the pandemic, but it also ensures that the existing facility plan will not face large-scale changes affecting the operation of medical institutions. The same flexibility principle also applies to other mobile epidemic prevention medical stations.

In Taiwan, thanks to the mobile swabbing rooms, medical institutions were able to maintain daily medical practice while conducting additional COVID-19 screening work at the same time. For instance, a hospital in Taipei experienced a nosocomial infection in January 2021; five containers of mobile swabbing rooms were set up immediately as screening stations and collected 600–800 specimens per day. During the outbreak in May 2021, the same hospital located in Taipei collected 10,000 specimens per month. A local clinic in Taichung set up a public expense PCR swabbing station authorized by Taiwan CDC on August 2021 and was able to collect 100 specimens per day with one swabbing container. A hospital located in Kaohsiung set up three containers as swabbing stations and collected 1500–1700 specimens per day. The screening capacity can be adjusted according to current needs. When the virus evolves into influenza in the future, a large number of screening stations will no longer be required, and the temporary mobile swabbing stations can be moved elsewhere when required.

### 4.2. Comparison with Other Mobile Facilities: Advantages and Disadvantages

Compared to two other mobile medical facilities designed and published on 2021, the Smart Pod mobile clinic [33] and the mobile eye hospital [34], the advantages and disadvantages of the mobile medical station of this study are presented and discussed. A Smart Pod is a mobile container unit built to participate in the community-based management of COVID-19 in the US in response to the burgeoning need for testing and vaccine delivery. The Smart Pod mobile clinic was deployed to provide primary care and COVID-19 testing to migrant farm workers in Iowa and disadvantaged Baltimore neighborhoods, monoclonal antibody infusion to patients in nursing homes and long-term care facilities and served the dual purpose of everyday community-based care and emergency mobilization. The mobile eye hospital was designed using established commercially available structures: an expandable shipping container and a rapid assembly field tent were included. The design emphasized providing comprehensive high-volume services without needing the logistical complexity of military operations. Therefore, focusing on easy assembly, dismantling and operation by minimal numbers of personnel. This design planned to provide governments and nongovernmental organizations with services to complement current eye health provisions in resource-scarce environments, offering a mobile surgical solution to rural and inaccessible areas.

The design concept of the Smart Pod is similar to our mobile medical station since both mobile facilities were designed in response to the COVID-19 pandemic. The similarities are as follows: (1) both were designed with deployable, expandable shipping containers; (2) they can be applied as laboratories of biosafety level (BSL) 2 and BSL 3; (3) they can be renovated into positive and negative air pressure rooms; (4) mobile clinics can provide vaccines, diagnosis and healthcare education. Although the mobile eye hospital was designed especially for eye care, there are still many similarities: (1) using commercially available containers as the main structure; (2) the shipping container is capable of being delivered by trucks; (3) all equipment is of Centers for Disease Control and Prevention (CDC) air exchange standards; (4) a positive pressure airflow system, air handling unit with high-efficiency particulate air (HEPA) and UV germicidal filtration system are incorporated; (5) both contain a back-up generator.

The similarity of the Smart Pod, the mobile eye hospital and the mobile medical station of this study show that our design is indeed sufficient to meet social needs, especially in terms of COVID-19 epidemic prevention awareness, creating a safe and comfortable environment that best suits medical staff, patients and the public. With high mobility, our mobile medical station can be customized according to local needs by either renovating the container space or adding the required equipment. The differences that remind us of the future direction in which we can strive to improve are as follows: (1) the Smart Pod mobile clinic is equipped with a barrier-free ramp so that disabled and wheelchair users can easily enter the mobile clinic. This is an important design that the mobile medical station of this study did not consider. The design of barrier-free space is the same as medical care, which is the focus of modern human rights, ensuring that everyone can move safely with ease to receive equal medical care. (2) In addition to being based in a container, the mobile eye hospital also uses field tents as a structure, which allows the eye care station to be expanded whenever required. This compound concept can also be applied to our mobile medical station to expand more possibilities if needed in the future.

### 4.3. Economic Value

To understand the practical application of mobile epidemic prevention medical stations from the perspective of medical management, it is necessary to consider whether the resources of the medical system can be used adequately and effectively. Among them, resource management needs to evaluate whether funds, costs, prices, equipment, materials, etc., are effectively used. From the standpoint of management, in order to operate effectively, reducing costs is the key factor of sustainable development. However, medical institutions’ social responsibility is to respond to the needs of the public. With the COVID-19 pandemic on the rise, the related equipment costs will inevitably increase. The rapidly changing nature of the pandemic should be taken into account when evaluating equipment costs and related resource investment. The future development of the pandemic is difficult to predict. Therefore, when planning and adjusting epidemic prevention facilities and materials, medical managers need highly flexible strategies in order to prevent resources from being wasted due to inappropriate use.

The current epidemic prevention strategies in Taiwan require a large number of screening operations and PCR tests; thus, mobile epidemic prevention medical stations are used as swabbing stations. The income of screening fees and variable cost of consumable materials is able to achieve an economic cycle. In Taiwan, the cost of PCR screening varies among medical institutions; the price range is between NTD 3500 (about USD 117) and NTD 5000 (about USD 167). The prices are also adjusted according to the severity of the pandemic, the universality of rapid test kits and the urgency of standard or priority. Taking a hospital located in central Taiwan as an example, since the swabbing station was established in October 2020, 1500 people have been screened each month, costing NTD 3500 per person, which makes more than NTD 5 million (about USD 175,000) per month. The cost of setting up a mobile positive swabbing station is about NTD 1 million. After deducting the cost of setting up from the income of screening fee, the remaining resources can be used to purchase other medical equipment and related supplies to provide better medical services to the public, forming an economic cycle to manage the balance between expenses and income.

### 4.4. Difficulty and Future Possibility

The main design concept of the mobile epidemic prevention medical station is to adjust its equipment and facilities according to local needs. Therefore, overcoming the obstacles encountered during design and actual construction becomes a challenge with great difficulty. For instance, the construction limitation in Palau was to solve the problem of facing infrastructure inconsistencies in the water and electricity supply. In dealing with on-site needs, the design and construction team must find solutions with interdisciplinary abilities other than the abilities of medical professionals.

In the future, even if the pandemic eases, the number of screening and isolation stations will need to decrease; however, the mobile stations can still be used for other functions. With new ideas and techniques, the containers can be turned into healthcare stations for rural areas and offshore islands or long-term community care stations, as mentioned in the design concept. Therefore, the mobile stations will not become idle equipment. Moreover, with the rapid development of science and technology, the application of AI-assisted diagnosis, telemedicine, big data (BD), machine learning (ML), medical imaging (MI) and computer-aided diagnosis (CAD) systems is predicted to effectively improve the ability of the mobile medical stations in the future.

## 5. Conclusions

Designing mobile epidemic prevention medical stations for COVID-19 was conducted to maintain medical capacity under limited resources. In this way, appropriate medical care can be provided to COVID-19 patients under the safety of quarantine. Suspected cases and screening procedures can also be taken care of without worrying about nosocomial infection. Last but not least, the mobility and flexibility of the design and its economic value reduce the risk of equipment investment for medical institutions, which plays an important role in considering medical sustainability.

## Data Availability

Publicly available datasets were analyzed in this study. This data can be found here: https://sites.google.com/cdc.gov.tw/2019-ncov/taiwan accessed on 16 February 2022.
